# Enhanced external counterpulsation, focusing on its effect on kidney function, and utilization in patients with kidney diseases: a systematic review

**DOI:** 10.2478/abm-2023-0062

**Published:** 2023-10-26

**Authors:** Thana Thongsricome, Weerapat Kositanurit, Sarawut Siwamogsatham, Khajohn Tiranathanagul

**Affiliations:** 1 Department of Physiology, Faculty of Medicine, Chulalongkorn University, Bangkok 10330, Thailand; 2 Clinical Research Center, Research Affairs, Faculty of Medicine, Chulalongkorn University, Bangkok 10330, Thailand; 3 Division of Nephrology, Department of Medicine, Chulalongkorn University, Bangkok 10330, Thailand

**Keywords:** cardiorenal syndrome, contrast induced nephropathy, cardiovascular disease, enhanced external counterpulsation, hemodialysis, renal function

## Abstract

**Background:**

Enhanced external counterpulsation (EECP) is provided by a noninvasive device positively affecting cardiovascular function via mechanisms called diastolic augmentation and systolic unloading. The renal aspects of EECP therapy have not been extensively investigated.

**Objectives:**

To assess the effect of EECP on renal function and to determine the application in patients with kidney disease.

**Methods:**

MEDLINE, EMBASE, SCOPUS, and Cochrane CENTRAL databases were searched for all studies involving EECP treatments. The title and abstract of all searched literatures were screened, and those focusing on renal outcome or conducting in kidney disease patients were selected.

**Results:**

Eight studies were included in the qualitative analysis. EECP increases stroke volume, mean arterial pressure, renal artery blood flow, renal plasma flow, glomerular filtration rate (GFR), plasma atrial natriuretic peptide, urine volume, and urinary sodium chloride excretion, but reduces the plasma concentration of renin and endothelin-1 in healthy subjects. A single session of EECP after radioactive contrast exposure could provide increased contrast clearance, and this reduces contrast-induced kidney injury in patients, irrespective of previous kidney function. Thirty-five-hour sessions of EECP treatment were illustrated to increase long-term estimated GFR in patients with chronic angina and heart failure. In cirrhotic patients, EECP fails to improve GFR and renal vascular resistance. EECP device could maintain blood pressure, decrease angina symptoms, and increase cardiac perfusion in hemodialysis patients.

**Conclusion:**

EECP treatment potentially increases renal perfusion and prevents kidney injury in several conditions. EECP possibly provides beneficial effects on hemodynamics and cardiac function in hemodialysis patients.

Enhanced external counterpulsation (EECP) is a therapy provided by a noninvasive device, which was initially utilized to maintain hemodynamic stability in patients with cardiac dysfunction for more than half a century [1]. Before the introduction of EECP, Kantrowitz et al. [1] introduced an intra-aortic balloon pump (IABP) in 1968, which is still used nowadays, for patients with cardiogenic shock and post-cardiac surgery. However, due to its invasive nature, IABP has been associated with several complications which may lead to significant mortality and morbidity, such as trauma to blood vessels, arterial embolization, hemolysis, lower limb ischemia, and device-associated infection [2]. For this purpose, EECP was introduced as a noninvasive and safer alternative to IABP, initially as EECP using a water-filled cylinder system, and then it was replaced with a more effective air-filled EECP in 1980–1983 [3, 4] (**Figure 1**). The device consists of 2–3 inflatable pneumatic cuffs applied around the thighs, legs, and buttock that can inflate or deflate synchronously with electrocardiography (ECG) waveform. Similar to the mechanism of IABP, EECP enhances cardiac function in both the systolic and diastolic phases [5, 6]. In the systolic phase, the fully inflated pneumatic cuff of EECP deflates rapidly in all parts to decrease systemic vascular resistance or afterload. This leads to suctional force for increasing stroke volume and decreasing the left ventricular oxygen demand. During the diastolic phase, the cuff inflates sequentially from the distal leg to the proximal thigh and buttock to increase venous return and central aortic blood pressure, so-called ‘diastolic augmentation’. This increasing diastolic blood pressure (DBP) results in increased cardiac perfusion. Perfusion to other organs may also be augmented to a lesser extent [5, 6].

**Figure 1.**
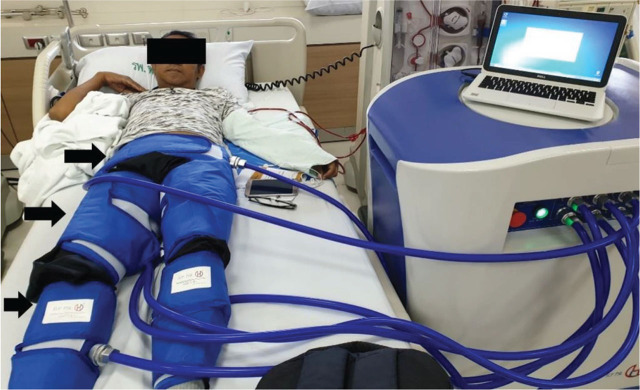
Application of EECP in King Chulalongkorn Memorial Hospital. Each arrow represents the cuff component in a 3-cuff EECP system (with consent from the patient for publication). This is a novel utilization of the device during the hemodialysis session to improve intradialytic hemodynamics. EECP is traditionally applied in subjects not receiving dialysis. EECP, enhanced external counterpulsation.

EECP has been demonstrated in many cardiovascular diseases, at least in part, due to reported positive cardiovascular effects of EECP in humans such as increasing myocardial perfusion and systolic function, improving endothelial function via a change in nitric oxide and endothelin-1 from shear stress generated from the counterpulsation, promoting angiogenesis of collateral coronary vessels, enhancing peripheral effects, and improving neurohormonal factors, all of which are similar to those from regular physical exercise [7,8,9]. There have been recognized benefits of EECP in ameliorating symptoms and improving the quality of life in patients with ischemic heart disease, congestive heart failure, and acute ischemic stroke [10, 11]. In addition, there have been studies investigating the beneficial effects of EECP in patients with central retinal artery occlusion and erectile dysfunction [12, 13]. Adverse effects from EECP therapy in previous studies are infrequent and mostly minor, such as skin blebs and muscle pain around the compression area, both of which are self-remitted [6, 10]. This makes EECP to be approved by the US Food and Drug Administration for refractory angina and congestive heart failure [9,10,11]. The major contraindications for EECP in most studies are aortic regurgitation and abdominal aortic aneurysm [6].

As stated above, EECP might potentially provide beneficial effects on renal hemodynamics and renal function. There have been certain available studies regarding this interesting issue. The present systematic review was conducted to determine the effect of EECP on renal function, and application of EECP in patients with kidney diseases.

## Materials and methods

This systematic review was conducted according to the Preferred Reporting Items for Systematic Reviews and Meta-Analyses (PRISMA) guidelines [14].

### Search strategy

As studies of EECP therapy in general, and those focusing on renal outcome or studying in kidney disease patients are scarce, we chose a less strict searching strategy to cover all possibly relevant studies. We searched the MEDLINE, EMBASE, SCOPUS, and Cochrane CENTRAL databases from inception until February 2023 using the following key search terms: “enhanced external counterpulsation” or “enhanced external counter-pulsation” or “enhanced external counterpulsation” or EECP. In addition, references in the selected studies were screened manually for additional records.

### Selection criteria

The title and abstract of all retrieved studies were independently screened and selected by two authors (TT and WK) and only studies focusing on renal outcome or kidney disease patients were selected. All disagreements were resolved by discussion and consensus with the third author (KT). EECP application of any protocols and durations, short- to long-term, was included and stated in the study details. The literature without a full text in English was excluded. Studies with reported quantitative outcomes and comparison groups were included for meta-analysis.

### Data extraction

The following data were extracted from the included studies: first author's name, year of publication, country of study, study design and comparison, included patients, study sample size, protocol, duration of EECP therapy along with data collection time, outcomes, measurements, contraindication for EECP, reported data, and adverse effects from the therapy.

### Quality assessment

The process of quality assessment was similar to that for data screening and selection. The quality of the included literature was evaluated with Methodological Index for Non-Randomized Studies (MINORS) if there was no comparison group. There were 8 items for non-comparative studies and each item was scored 0 if not reported; 1 when reported but inadequate; and 2 when reported and adequate. The global ideal score was 16 for non-comparative studies. However, there was no validated cut point for the score [15]. For randomized controlled trial, the Cochrane Collaboration's tool for assessing risk of bias was applied [16].

### Statistical analysis

As the included studies could not be combined into a meta-analysis due to their lack of comparison group and difference in measured outcomes, our systematic review is presented mainly in a narrative manner and no statistical analysis was utilized.

## Results

**Figure 2.**
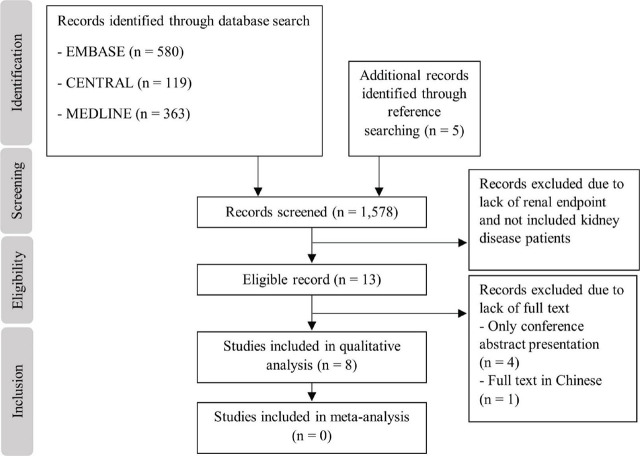
Flowchart of study selection.

The flowchart of study selection is depicted in **Figure 2**. From an initial set of 1,578 records, 5 of 13 eligible studies were further excluded due to lack of full text to yield the final 8 studies for qualitative analysis. There was no study for meta-analysis due to their lack of similar qualitative outcomes and comparison groups. The characteristics of the included studies are detailed in **Table 1**. The MINORS quality assessment of the studies is demonstrated in **Table 2**. For an included randomized controlled trial, the Cochrane Collaboration's tool for assessing risk of bias reveals low risk of selection bias, detection bias, attrition bias, and reporting bias due to a clear explanation on randomization and low number of loss follow-up. There was no declaration about the allocation concealment. Despite a non-sham control manner, the performance bias was considered as low risk because all outcomes were objective laboratory measurements.

**Table 1.** Characteristics of the included studies

**Study**	**Country**	**Design**	**Included patients**	**Sample size (n)**	**EECP therapy**	**Contraindication for EECP or exclusion criteria**	**Outcome and measurement**
Applebaum et al. [17]	United States and India	Pre- and post-procedure comparison (no control group)	Atherosclerotic heart disease, age 55 ± 8 years	18 (male, 78%)	- Machine: Cardiomedics, Inc., Irvine, California - 2 flexible cuffs - Cuff pressure up to a point at which the peak diastolic pressure wave reached the height of the systolic pressure wave on the finger plethysmography (150–180 mmHg for most patients) - 1 session, duration 30 min	- History of recent lower extremity thrombophlebitis, severe ischemia, or trauma including surgical incision and amputation - Moderate to severe aortic regurgitation - Severe congestive heart failure - Uncontrolled hypertension - Uncontrolled arrhythmia - Thrombolytic or anticoagulation agents use	Renal artery blood flow, measured every 5 minutes during and immediately after the counterpulsation with duplex ultrasonography (angle correction of ≤60°)
Werner et al. [18]	Germany	Pre- and post-procedure comparison (no control group)	Healthy volunteers, age 28 ± 4 years	16	- Machine: Vasomedical Inc., Westbury, New York, and Cardiomedics Inc., Irvine, California - 2 cuffs (calves and thighs) - Cuff pressure of 200 mmHg - 1 session, duration 1 h	No available data	Changes in flow volume in carotid, vertebral, hepatic, renal, and internal iliac arteries, measured by duplex ultrasonography
Werner et al. [19]	Germany	Pre- and post-procedure comparison in (1) cirrhotic patients and (2) healthy subjects	- Cirrhotic patients diagnosed by hepatologists, age 54.4 ± 10.5 years - Healthy subjects, age 23.7 ± 2.5 years	16 cirrhotic patients and 12 healthy subjects (male, 50%)	- Machine: Vasomedical Inc., Westbury, New York - 2 cuffs (calves and upper thighs) - Cuff pressure of 250–300 mmHg - 1 session (performed in the early afternoon), duration 2 h	- Aortic regurgitation - Aortic aneurysm - Atrial fibrillation - Deep venous thrombosis, leg ulcer, marked peripheral edema - INR >2 (Screened by electrocardiogram, echocardiogram, and duplex sonography of the lower extremities)	- GFR by inulin clearance (continuous infusion) - Renal plasma flow by aminohippurate sodium clearance (continuous infusion) - Continuous radial artery blood pressure, monitored by vascular unloading technique - Plasma concentrations of endothelin-1, measured by ELISA kit - Plasma concentrations of renin, ANP, ADH, epinephrine and N-epinephrine, measured by radioimmunoassay - Urinary volume determined every 30 min - Urinary excretion rates of sodium and chloride, measured by flame photometry - Urinary osmolality, measured by freezing point depression
Onuigbo [20]	United States	Case series	Hemodialysis patients with IDH and hypoalbuminemia refractory to conventional treatments	3	Using sequential compression device as a mini-EECP - Machine: Flowtron Excel deep venous thrombosis prophylaxis system (Huntleigh Healthcare, Poland) - Cuff pressures of 40 mmHg - The cuff is applied to the calves throughout the hemodialysis session, and inflation of the cuffs is alternated between both calves every other minute.	Not stated	- Achieved ultrafiltration volume in the dialysis sessions - Patient tolerability and IDH episodes
Ruangkanchanasetr et al. [21]	Thailand	Longitudinal pre- and post-procedure comparison (no control group)	Age ≥18 years with chronic stable angina and/or heart failure	30 (male, 76.7%, chronic angina 76.7%, heart failure 23.3%)	- Machine: Vasomedical Inc., Westbury, New York - 35 sessions of 1-h daily EECP treatment over a period of 7–8 weeks - Cuff pressure and amount: not specify	- Unstable angina, acute myocardial Infarction, decompensated heart failure in the preceding one month - Undergoing coronary angiography or coronary artery bypass grafting in the preceding 1 month - Blood pressure >180/110 mmHg - Severe symptomatic peripheral vascular disease - GFR <15 mL/min/1.73 m^2^	- Serum creatinine, measured by enzymatic methods - Serum cystatin C, measured by particle-enhanced immunonephelometric assay - Estimated GFR using combination of serum creatinine and cystatin C - NT-proBNP, measured by a sandwich immunoassay - Non-invasive blood pressure measurement (The median follow-up time after starting EECP treatment was 16 months)
Wu et al. [22]	Taiwan	Longitudinal pre- and post-procedure comparison (no control group)	Hemodialysis patients with coronary artery disease and angina refractory to medical treatment and unable or unwilling for revascularization	36 (male, 61.3%)	- Machine: Vasomedical Inc., Westbury, New York - Duration of 1- or 2-h daily EECP treatment for 5 days per week to reach total of 35 h - Cuff pressures of 260–300 mmHg to achieve mean peak diastolic augmentation of 1.6	- Significant aortic regurgitation - Abdominal aortic aneurysm (Screened by echocardiography and abdominal sonography)	- Angina symptom, measured by Canadian Cardiovascular Society Angina Grading scale - Angina medications - Myocardial perfusion, assessed by Thallium-201 imaging with pharmacological stress - Cardiovascular events (Assess immediately after complete 35 h of EECP and 1 year after complete the therapy)
Zhang et al. [23]	China	Randomized, non-sham-controlled	Age >18 years undergoing a diagnostic contrast-enhanced computed tomography with estimated GFR using CKD-EPI of 60–89 mL/min/1.73 m^2^	121 (male, 62%, hypertension 56%, diabetes 27%)	- A 1-h session of EECP therapy at 2 h after exposure to the contrast media - Target diastolic/systolic augmentation ratio of 1.0–1.2	- Blood pressure >180/100 mmHg - Hemorrhagic disease or bleeding tendency including INR >2 - Uncontrolled tachyarrhythmia - Severe aortic insufficiency - Acute heart failure - Arterial dissection or aneurysm - Lower-extremityvenous thrombosis - Infection, pregnancy, thyroid disease, tumor - Recent exposure to contrast media or nephrotoxic drugs	- Increase of serum cystatin C ≥10% at 24^th^ h after contrast exposure - Iopromide contrast clearance measurement using plasma concentration of iopromide at 2^nd^, and 3^rd^ h - Conventional diagnosis of contrast-induced kidney injury using serum creatinine concentration at 48^th^ h - Adverse clinical events
Zeng et al. [24]	China	Prospective cohort, compared with active comparator (standard dose of 0.9% NaCl hydration)	Age ≥18 years with estimated GFR <60 mL/min/1.73 m^2^ not on dialysis; Receiving coronary angiography and percutaneous intervention	230 (male, 76%, diabetes 36.1%, hypertension 77%, mean estimated GFR 42 mL/min/1.73 m^2^)	A once daily 1-h session of EECP therapy at 24 h before and 48–72 h after the intervention	(1) patients who had used iodinated contrast medium 30 d before inclusion, (2) patients with AKI due to other clear causes, (3) patients requesting withdrawal, (4) patients who failed to receive the re-examination of renal function indicators on time after surgery, (5) patients who underwent hemodialysis within 48 h after surgery, and (6) patients with uremia who received long-term hemodialysis.	Serum creatinine increase ≥0.3, ≥0.5 mg/dL or ≥25% relative to baseline value within 48–72 h after iodinated contrast exposure

ADH, antidiuretic hormone; ANP, atrial natriuretic peptide; CKD-EPI, Chronic Kidney Disease Epidemiology Collaboration equation; EECP, enhanced external counterpulsation; ELISA, Enzyme-Linked Immunosorbent Assay; GFR, glomerular filtration rate; IDH, intradialytic hypotension; INR, international normalized ratio; NT-proBNP, N-terminal pro b-type natriuretic peptide.

**Table 2.** MINORS quality assessment of the included studies Enhanced external counterpulsation and kidney

	**Applebaum 1997**	**Werner 1999**	**Werner 2005**	**Onuigbo 2013**	**Ruangkanchanasetr 2013**	**Wu 2014**	**Zeng 2022**
A stated aim of the study	2	2	2	2	2	2	2
Inclusion of consecutive patients	1	1	1	0	2	2	2
Prospective collection of data	2	2	2	2	2	2	0
Endpoint appropriate to the study aim	2	2	2	2	2	2	2
Unbiased assessment of endpoints	1	1	1	0	1	1	2
Follow-up period appropriate to the major endpoint	2	2	1	2	2	2	1
Loss to follow-up not exceeding 5%	2	2	2	2	2	0	2
Prospective calculation of the study size	0	0	0	0	2	0	1
Total	12	12	11	10	15	11	12

The items are scored 0 (not reported), 1 (reported but inadequate), or 2 (reported and adequate).

MINORS, Methodological Index for Non-Randomized Studies.

### Effects of EECP on renal physiology

**Figure 3.**
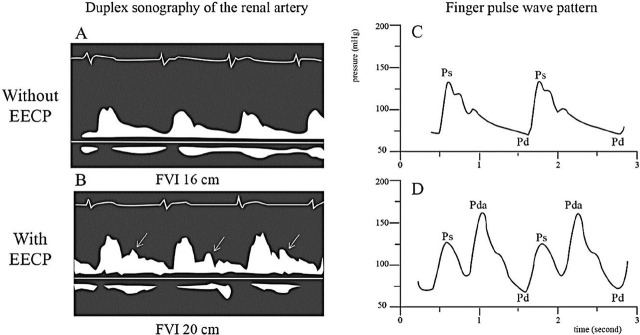
Duplex sonography of the renal artery without EECP **(A)**, compared with with EECP treatment **(B)** demonstrating augmented diastolic flow velocity (arrow), and finger pulse wave pattern without EECP **(C)**, compared with with EECP treatment **(D)** systolic blood pressure (Ps), diastolic blood pressure (Pd), and augmented diastolic blood pressure (Pda). Systolic pressure reduces after EECP treatment and results in decreased cardiac oxygen demand. The FVI measured from duplex sonography also increases upon EECP therapy, reflecting increased blood flow to the artery. The finger plethysmography waveform during the therapy is similar to that from pulse wave analysis. The figure is based on results demonstrated by Applebaum et al. [17]. EECP, enhanced external counterpulsation; FVI, flow velocity integral.

In healthy subjects, EECP therapy has an immediate effect on systemic hemodynamics, local renal hemodynamics, and neurohormonal factors [18, 19]. Stroke volume increased by 12 ± 9% (*P* < 0.05) upon application of EECP [18] with a trend in increased mean arterial pressure [19]. Even with increased stroke volume, the percentage of blood flow to many arteries, including abdominal aorta, common carotid artery, internal carotid artery, right and left vertebral arteries, hepatic artery, left coronary artery, internal iliac artery, and renal artery, increases to a higher degree. However, there was no significant change in arterial diameters except for the abdominal aorta, whose diameter increased by 1.0 ± 0.8 mm upon diastolic augmentation. This blood flow to the right renal artery was increased by 21 ± 9% [18]. The enhanced blood flow in the internal carotid and left main coronary artery was even higher with the application of a current higher pressure 3-cuff system compared with the earlier 2-cuff system, but there was no study in the renal circulation [18]. This blood flow augmentation was also confirmed in patients with atherosclerotic heart disease, whose increment in renal artery flow velocity integral was 19% (*P* = 0.0001) along with a peak systolic flow velocity increase of 8% (*P* = 0.006) [17]. The augmentation can be monitored by duplex sonography waveform in all affected arteries, pulse wave analysis, or plethysmography waveform mostly applied in the finger during the EECP session (**Figure 3**). The cardiac cycle lengths were not affected by EECP therapy. Of interest, these renal and systemic augmentation were fully reversible after the termination of EECP session [17].

In consort with the systemic hemodynamic changes, renal hemodynamic parameters in healthy subjects as measured by indicator clearance technique also exhibited increased renal plasma flow (515 ± 134 to 676 ± 189 mL/min, *P* < 0.05), glomerular filtration rate (68 ± 16 to 84 ± 22 mL/min/1.73 m^2^, *P* < 0.05), and a trend in decreasing renal vascular resistance (68.5 ± 17.2 to 55.2 ± 12.5 mmHg·min/l) [19]. Moreover, hormonal changes in the short term after one EECP session showed significantly reduced plasma renin and endothelin-1 concentrations with significantly increased plasma atrial natriuretic peptide (ANP) concentrations. Urinary excretion rate was also increased by about 28% along, with about 50% increases in urinary sodium and chloride excretion rate. Urinary osmolality was significantly decreased from 487 ± 280 to 274 ± 69 mOsm/kg H_2_O (*P* < 0.05). Plasma epinephrine and norepinephrine concentrations were relatively unchanged [19].

### Renal outcomes of EECP in patients without advanced chronic kidney disease

#### Cirrhotic patients

Patients with cirrhosis are well known to be at risk of renal hypoperfusion from splanchnic blood redistribution, resulting in acute to chronic renal dysfunction, so-called hepatorenal syndrome. Werner et al. [19] demonstrated lower values of mean arterial pressure, renal plasma flow, urine volume, urinary sodium excretion, and urinary chloride excretion in a group of cirrhotic patients with mild renal impairment, whose mean glomerular filtration rate (GFR) of 70 mL/min, compared with healthy controls with a similar level of GFR. Cirrhotic patients also had elevated plasma levels of renin, ANP, antidiuretic hormone, endothelin-1, epinephrine, and norepinephrine. Application of EECP for one session could increase mean arterial pressure, urine volume, urinary sodium, and urinary chloride excretion, but significantly reduced plasma renin and increased plasma ANP concentration. However, the therapy could not significantly increase GFR and renal plasma flow nor reduce renal vascular resistance observed in the healthy subjects. Instead, renal vascular resistance increased significantly. Therefore, the increased sodium, chloride, and volume excretion could be secondary from increased systemic blood pressure, so-called pressure natriuresis, rather than the directly improved renal hemodynamics. Moreover, there was no study investigating the effect of EECP on renal physiology and parameters in both healthy and cirrhotic subjects in a longer duration than a single session [19].

#### Prevention of contrast-induced kidney injury

Zhang et al. [23] demonstrated in a randomized non-sham-controlled trial that a 1-h EECP session at the 2^nd^ h after iopromide radiocontrast exposure for diagnostic computed tomography significantly enhanced the contrast clearance as calculated from 2-point plasma iopromide concentration measurements without urine collection (59.92 ± 8.84 mL/min/1.73 m^2^ compared with that of 46.80 ± 9.26 mL/min/1.73 m^2^ in the control group, *P* < 0.001). Moreover, EECP therapy yielded a significant reduction in the incidence of ≥10% increase in serum cystatin C at the 24^th^ h after radiocontrast exposure (adjusted odds ratio of 0.235, 95% confidence interval of 0.085–0.653) [23]. This threshold of increased serum cystatin C was obtained by a previous study to be an early marker for contrast-induced kidney injury [25]. However, the majority of the studied subjects were at low risk of developing contrast-induced kidney injury, with Mehran risk score of 6–10 in 13.5%, and no subjects had the Mehran risk score of >10. The incidence of conventionally diagnosed contrast-induced kidney injury with serum creatinine measurement at the 24^th^ and 48th h after radiocontrast exposure was low in the study (none in the EECP group compared with 7% in the control group, *P* 0.042) [23].

Zeng et al. [24] explored this protective effect of EECP in patients with chronic kidney disease (CKD) with or without diabetes mellitus receiving intra-arterial contrast in coronary angiography procedure, who had high risk for contrast-induced kidney injury. Unlike the previous study, the authors applied two EECP sessions, one before the procedure and the other after radiocontrast exposure. The postoperative estimated GFR was increased in those receiving EECP therapy (44.0 ± 15.6, compared with 41.5 ± 12.7 mL/min/1.73 m^2^, *P* = 0.047). The incidence of contrast-induced kidney injury was significantly lower with EECP therapy (3.8%, compared with 16.7% in those receiving standard 0.9% NaCl hydration alone, *P*-value 0.042) along with lower high-sensitivity C-reactive protein, possibly reflecting reduced renal inflammation from contrast media [24].

### Cardiovascular disease patients with or without cardiorenal syndrome

Ruangkanchanasetr et al. [21] examined the long-term effects after a widely accepted protocol of 1-h daily EECP therapy for 35 sessions in 30 chronic angina or heart failure patients receiving optimal medical therapy. The study demonstrated significantly improved heart failure symptoms and estimated GFR derived from a combination of serum creatinine and serum cystatin C from 70.47 mL/min/1.73 m^2^ to 76.27 mL/min/1.73 m^2^ after a median follow-up time of 16 months after the treatment (*P*-value 0.006) with a trend in decreasing N-terminal pro b-type natriuretic peptide (NT-proBNP). A significant increase in estimated GFR was identified mainly in a subgroup of 12 patients with an initial estimated GFR of <60 mL/min/1.73 m^2^ and those with initial NT-proBNP of >125 pg/mL, probably representing patients with cardiorenal syndrome [21].

### Cardiovascular benefits of EECP in hemodialysis patients

Previously mentioned studies on renal outcomes of EECP included patients with relatively preserved renal function or early stage of CKD. In hemodialysis patients, Wu et al. [22] studied the long-term effect of a standard EECP treatment protocol in patients with coronary artery disease and refractory angina. The study demonstrated significant improvement both subjectively, by decreased angina Canadian Cardiovascular Society Angina Grading Scale since the completion of EECP treatment in 85% of the patients and up to 1 year after the treatment in 66%, and objectively, by complete or partial resolution from Thallium-201 myocardial perfusion imaging, in 65% of the 20 patients receiving the perfusion imaging. Diabetes mellitus and hyperphosphatemia were negative predictors of sustained effectiveness of EECP treatment on reducing angina symptoms. Of interest, two patients did receive repeated EECP treatment to relieve the angina symptom [22].

However, there has been no study applying EECP treatment during the dialysis session. There was only one case series by Onuigbo [20], who successfully utilized intrahemodialysis sequential compression device for deep vein thrombosis with much lower cuff pressure than EECP therapy to maintain hemodynamics in three patients with refractory intradialytic hypotension despite management with other measures such as cool dialysate, intravenous crystalloid solution, intravenous albumin, oral midodrine, anemia correction, and sodium profiling. Besides blood pressure and achieved ultrafiltration volume, there were no available measured hemodynamic parameters.

There is an ongoing randomized controlled trial conducted by the authors of this systematic review regarding application of EECP during hemodialysis. A preliminary result reveals the maintenance of cardiac output and DBP, which are normally declined due to the ultrafiltration process. The incidence of intradialytic hypotension is absent in the EECP group and the longer term effects on cardiovascular parameters are under investigation [26].

### Tolerability and side effects

Two patients in the study by Wu et al. [22] did not complete the EECP session due to intracranial hemorrhage in one patient and intractable hemorrhoid bleeding in the other [22]. Four patients had nonfatal myocardial infarction and five patients died during the follow-up period, but the association with EECP therapy was unknown due to lacking of control group and high cardiovascular risk in hemodialysis patients. Other included studies did not specify the incidence of adverse events. This necessitates closed surveillance upon further application of EECP in kidney disease patients. Other possible adverse events reported in the general population include paroxysmal atrial fibrillation, leg discomfort, fatigue of limb muscles, headache, skin bruises, and blistering which are self-remitted and not interfere with the treatment [6].

## Discussion

EECP has long been shown in patients with coronary artery disease to increase cardiac output, coronary blood flow, and myocardial perfusion via the diastolic augmentation and systolic unloading mechanisms [27,28,29]. These beneficial effects have been translated into numerous clinical trials with long-term cardiovascular endpoints and involving patients with various cardiac conditions [30]. Regarding renal function, EECP has been shown to increase renal blood flow and GFR along with decreased renal vascular resistance, likely from diastolic augmentation, in healthy subjects after a single session of therapy [17,18,19]. Moreover, hormonal changes that are analogous to hypervolemic state have been evidenced. These include decreases in plasma renin, endothelin-1, and urinary osmolality, but increases in plasma ANP, urine volume, urinary sodium excretion, and urinary chloride excretion [19]. However, these benefits may be temporary and reversible after finishing the EECP treatment.

To date, there have been a few studies investigating the renoprotective effect of EECP, especially those with treatment duration of more than a single EECP session. The most promising renoprotective effect of EECP is the prevention of contrast-induced kidney injury. A recent randomized controlled study by Zhang et al. [23], which included patients with relatively preserved renal function and at low risk of contrast-induced kidney injury, demonstrated an enhanced radiocontrast clearance in parallel with the increased GFR with a single session of EECP therapy. The study may be underpowered due to the low incidence of kidney injury. Another non-randomized study by Zeng et al. [24] lately investigated patients in a more realistic manner by including patients at high risk for contrast-induced kidney injury, such as diabetes mellitus, or CKD, undergoing coronary intervention, and repeating the second EECP session after contrast exposure. The similar renoprotective effect of EECP found in this study strengthened the potential benefit of EECP in preventing contrast-induced kidney injury in various patient subgroups.

Regarding patients with cardiorenal syndrome, a study by Ruangkanchanasetr et al. [21] applying a standard 35-h EECP protocol for 16 months demonstrated a significant improvement of estimated GFR of 5.8 mL/min/1.73 m^2^, especially in those with initial estimated GFR of <60 mL/min/1.73 m^2^ and high initial NT-proBNP level. This possible amelioration of progressive cardiorenal syndrome by EECP is limited by the study design that had no control group. As such, the change in estimated GFR might be mediated by other causes such as the heart failure treatment [21]. In cirrhotic patients, a study by Werner et al. [19] failed to show improvement in renal plasma flow and GFR despite reduced plasma renin and increments in blood pressure, plasma ANP, urine volume, urinary sodium excretion, and urinary chloride excretion. Therefore, a single session of EECP therapy could not completely ameliorate the hepatorenal syndrome and the effect of longer-term EECP therapy requires further studies.

In hemodialysis patients, Wu et al. [22] demonstrated the benefits of EECP in reducing angina symptoms for up to 1 year and increasing cardiac perfusion measured by thallium imaging in refractory angina patients, like those found in patients without CKD. This might result from prevention of cardiac output and blood pressure decline that normally occur during hemodialysis, which may decrease organ perfusion [26]. However, additional cardiovascular risk factors unique to CKD population such as hyperphosphatemia and vascular calcification might reduce the efficacy of many treatments, including EECP in preventing cardiovascular event in these patients [22, 31]. In this regard, the long-term cardiovascular benefit of EECP in CKD patients may be less obvious than that in non-CKD population, and this issue should be investigated in larger clinical trials. This beneficial hemodynamic effect of EECP during hemodialysis may also be utilized to ameliorating refractory intradialytic hypotension in adjunct with all standard therapies [20].

In terms of adverse effects, EECP application in the general population is a relatively safe treatment modality after appropriate patient selection and exclusion of contraindications such as significant aortic regurgitation and abdominal aortic aneurysm. Possible adverse effects to be monitored include intracranial hemorrhage, hemorrhoid bleeding, headache, minor muscular pain, and cutaneous irritation. However, the adverse effects of EECP application in patients with kidney disease have been rarely reported and require further surveillance.

Despite being the first study to comprehensively review the application of EECP in patients with kidney disease, the limitation of our study is a few included studies. This also impedes the author from conducting meta-analysis. Moreover, the varied quality assessment of the included studies necessitates more mechanistic and clinical studies with good study design, larger sample size, more various patient subgroups, adequate follow-up time, and hard endpoints before the final consensus on this topic could be drawn.

In conclusion, EECP therapy is safe and potentially able to prevent renal injury in patients with cirrhosis, heart failure, radiocontrast exposure, and chronic angina. In hemodialysis patients, EECP possibly poses a positive cardiovascular effect. However, closed surveillance for possible additional adverse effects after application of EECP in this group of patients is required.
